# The Hepatic Imposter: Solitary Necrotic Nodules Masquerading as Hepatic Metastasis

**DOI:** 10.7759/cureus.59154

**Published:** 2024-04-27

**Authors:** Mohamed A Ebrahim, Eli A Zaher, Parth Patel, Omar Al Salman, Konrad Stelmark

**Affiliations:** 1 Internal Medicine, Ascension Saint Joseph Hospital, Chicago, USA; 2 Gastroenterology, Ascension Saint Joseph Hospital, Joliet, USA; 3 Internal Medicine, Poznan University of Medical Sciences, Poznań, POL

**Keywords:** computed tomography, hepatocellular carcinoma (hcc), metastasis, necrosis, hepatic nodules

## Abstract

Solitary necrotic nodules in the liver present a diagnostic challenge due to their rarity and resemblance to metastatic tumors. We report a case where imaging revealed multiple hepatic lesions suggestive of malignancy, prompting a needle biopsy. Histopathology confirmed necrosis without malignancy. Despite advancements in imaging modalities, distinguishing solitary necrotic nodules from metastases remains difficult. Recognition of characteristic imaging features and consideration of biopsy are crucial for accurate diagnosis and management. This case underscores the importance of thorough evaluation and differential diagnosis in liver lesions to prevent unnecessary surgical interventions and ensure appropriate clinical care.

## Introduction

Necrotic hepatic nodules are rare benign lesions featuring a central necrotic core surrounded by a fibrotic capsule. Various theories about its origin have been proposed, including potential trauma, prior parasitic infection, or sclerosing hemangiomas. These nodules are often located beneath the liver capsule, slightly protruding from its surface, with distinct boundaries, occasionally leading to misinterpretation as metastatic growths [1]. Clinicians face challenges in accurate diagnosis due to the absence of distinctive clinical or radiological features [2,3]. Herein, we present a case of multiple necrotic hepatic nodules initially suspected to be of primary hepatic or metastatic origin in an elderly individual.

## Case presentation

A 76-year-old obese male with a history of chronic kidney disease presented to the emergency department with complaints of intractable non-bloody vomiting and generalized abdominal pain for the preceding two weeks. He never had similar symptoms in the past and did not notice any relieving or exacerbating factors. No fevers or chills were reported. He denied using medications or illicit substances. He is a former 40-pack-year smoker and an occasional drinker. 

Upon admission, vital signs were within normal limits. Physical examination was unremarkable. Blood work-up was consistent with an acute kidney injury, but was otherwise negative (Table 1).

**Table 1:** Blood work upon admission The patient's baseline creatinine is around 2 mg/dL

Component	Result	Reference range
Hemoglobin (g/dL)	14.0	12.0-15.3
White cell count (k/mm cu)	9.0	4.0-11.0
Mean corpuscular volume (f/L)	90	80.0-100.0
Platelets (k/mm cu)	300	150-450
Creatinine (mg/dL)	3.5	0.6-1.2
Sodium (mmol/L)	135	133-144
Albumin (g/dL)	4.0	3.5-5.7
Aspartate aminotransferase (IU/L)	30	13-39
Alanine aminotransferase (IU/L)	42	7-52
Total bilirubin (mg/dL)	0.5	0.0-1.0
Alkaline phosphatase (IU/L)	100	35-104
International normalized ratio	1.0	0.9-1.1
Lipase (IU/L)	20	11-82

**Figure 1:**
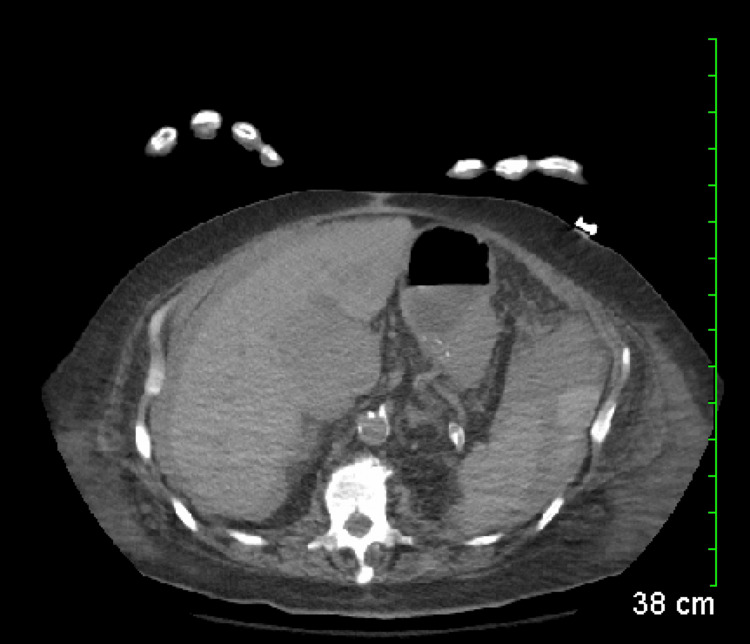
CT of the abdomen without contrast Multiple indeterminate low-density hepatic lesions, the largest measuring 7.5 cm. There is likewise moderate volume ascites seen

CT of the abdomen without contrast showed multiple low-density hepatic lesions suspicious for a metastatic or primary tumor (Figure 1). 

**Figure 2:**
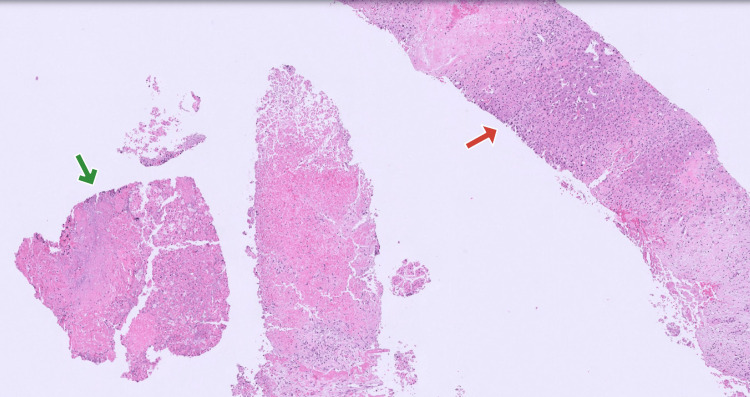
Liver histology Red arrow pointing towards an area of normal hepatic histology. Green arrow pointing towards an area of hepatic necrosis

The patient was up to date on his colonoscopy screening, which is normal. He refused to have a repeat colonoscopy during the admission. Interventional radiology performed an ultrasound-guided needle biopsy of the liver, which showed necrosis without evidence of malignancy (Figure 2).

Alpha-fetoprotein, carcinoembryonic antigen, and hepatitis panel were likewise negative. The patient recovered well with supportive management and was discharged home in good health.

## Discussion

A solitary necrotic nodule in the liver is a rare finding during ultrasound or CT scans and is often mistaken for necrotic metastasis [3]. Despite its singular name, this type of nodule can sometimes be multiple [4]. In many instances, it signifies the final stage of various benign conditions like larval infestation, sclerosed hemangioma, or trauma. Under the microscope, the nodule typically comprises a fibrotic outer layer with inflammatory cells surrounding a central core of necrotic material. These nodules can appear beneath the liver's outer covering or within its deeper tissues [5]. 

Solitary necrotic nodules, classified as benign lesions with no documented instances of severe complications like malignant transformation, pose a diagnostic challenge. Therefore, it's crucial for radiologists to recognize the imaging characteristics associated with solitary necrotic nodules of the liver to prevent unnecessary surgical interventions. Solitary necrotic nodules in the liver typically manifest as small, well-defined lesions measuring under 3.0 cm, displaying a round, oval, or lobulated morphology [2,6]. Predominantly situated in the right lobe and often found in the liver's subcapsular regions, these nodules are primarily solitary, although rare cases of multiple lesions within one patient have been documented, as observed in this particular case [6].

Recent advancements in liver surgery have resulted in an increased number of individuals eligible for partial hepatic resection due to metastatic diseases. Imaging plays a pivotal role in providing detailed maps of hepatic metastases and aiding in the careful selection of patients before surgery, thus reducing unnecessary surgical exploration. Non-invasive imaging methods such as ultrasound, CT, and MRI have recently shown improvements in detecting hepatic metastases. Among these, CT arterial portography (CTAP) is recognized as the most sensitive for detecting focal liver lesions, although its high false-positive rate is seen as a drawback [7]. Intraoperative ultrasound has also been utilized to uncover small metastases not detectable through direct palpation or other preoperative imaging methods. Nevertheless, all of these radiological features of these modalities lack the specificity required for accurately characterizing liver tumors [8]. In our case, the radiological observations of the multiple necrotic nodules in the liver resembled those of hepatic metastasis. Distinguishing between solitary necrotic nodules and hepatic metastasis using any imaging modality is highly challenging, if not impossible, due to their similar features, including central hypoattenuation caused by necrosis and peripheral hyperattenuation attributed to fibrosis. These similarities underscore the need for a more precise diagnostic approach. As such, a needle biopsy becomes indispensable for achieving an accurate diagnosis in such cases.

## Conclusions

Solitary necrotic nodules in the liver can appear as either single or multiple lesions on radiologic imaging, potentially leading to misinterpretation as metastatic hepatic tumors. The diagnostic challenge posed by solitary necrotic nodules in the liver highlights the importance of thorough evaluation and differential diagnosis during radiologic imaging. Their resemblance to metastatic hepatic tumors highlights the need for careful consideration and, when necessary, further confirmatory testing to ensure accurate diagnosis and appropriate clinical management.
